# Immediate impacts of COVID-19 pandemic on bean value chain in selected countries in sub-Saharan Africa

**DOI:** 10.1016/j.agsy.2020.103034

**Published:** 2021-03

**Authors:** Eileen Bogweh Nchanji, Cosmas Kweyu Lutomia, Rowland Chirwa, Noel Templer, Jean Claude Rubyogo, Patricia Onyango

**Affiliations:** a International Center for Tropical Agriculture, Kenya; b Kenya Agricultural and Livestock Research Organization, Kenya; c International Center for Tropical Agriculture, Malawi

**Keywords:** COVID-19, Bean value chain, Sub-Saharan Africa, Agriculture, Food system

## Abstract

Africa's agriculture and food systems were already grappling with challenges such as climate change and weather variability, pests and disease, and regional conflicts. With rising new cases of COVID 19 propelling various African governments to enforce strict restrictions of varying degrees to curb the spread. Thus, the pandemic posed unprecedented shocks on agriculture and food supply chains in Sub Saharan Africa. In this study, we use survey data collected from nine countries in Central, Eastern, and Southern, Africa to understand the immediate impact of COVID-19 on production, distribution, and consumption of common beans, and possible food security implications. Descriptive analysis of data collected from bean farmers, aggregators, processors, bean regional coordinators, and mechanization dealers reveal that COVID-19 and government restrictions had impacted the availability and cost of farm inputs and labour, distribution, and consumption of beans in Eastern and Southern Africa. The immediate impacts were dire in Southern Africa with Central Africa slightly impacted. The production and distribution challenges negatively impacted on frequency and patterns of food consumption in households in Africa. Thus, the pandemic poses a greater risk to food security and poverty in the region. Governments could play a significant role in supporting the needs of smallholder farmers, traders and other actors through provision of subsidized agricultural inputs.

## Highlights

• COVID 19 is redressing the agricultural gains in Africa.
• Building resilience of food systems is important.
• COVID 19 is exacerbating already existing agricultural challenges.
• Government incentive is crucial to cushion producers in a crisis.

## 1 Introduction

Food production in Sub Saharan Africa (SSA) is relatively low compared to the rest of the world. As a result, food insecurity is a persistent challenge in the region. Climate change, nutrient depleted soil, crop pests and diseases, frequent civil unrests and the outbreak of diseases such as the Spanish Flu, influenza and HIV/AIDs have contributed to low food production and distribution affecting agricultural activities in SSA prior to the emergence of the SARS-CoV-2 (COVID-19) pandemic (World Health Organization, 2020a). These pandemics have not only caused human suffering and death, but also reversed gains in the eradication of poverty and food insecurity to meet the Sustainable Development Goals 1 on Zero hunger.

When the first cases of COVID 19 were reported in Africa, Egypt in February, most countries in SSA closed their borders and shut down their air spaces to reduce the number of imported coronavirus cases. Other measures, including lockdowns, widespread restriction on movement, and a ban on public gatherings were put in place with varying degrees of strictness as shown in Table 1 below.

**Table 1:** Summary of public policies and measures to prevent the spread of coronavirus.

Country	Policies and containment measures
Kenya	Partial lockdown (May 7), partial border closure (May 16), cessation of movement in high-risk counties/regions (April 6), dusk to dawn curfews (March 29), social distancing and mandatory mask-wearing (April 6), closure of schools (March 15), closure of churches and non-essential businesses (April 11), restricted air transport (8 April), ban on travel across the Tanzanian border (16 May). UN Migration Agency (2020)
Uganda	Nationwide lockdown (March 31, eased June 2), curfew (March 31), closure of non-food selling businesses (March 31), and restricted transportation (March 31, enhanced April 10), social distancing and mandatory mask-wearing. UN Migration Agency (2020) and Anadolu Agency (2020).
Tanzania	Closure of schools, ban on public gatherings (17 March), advise encouraging people to avoid unnecessary movements. No formal internal movement restrictions. Suspension of air travel and intercountry public bus services (March 25, reinforced April 11, relaxed on May 14 and lifted on May 18), Kenya border closure (May 17). UN Migration Agency (2020) and Kell (2020).
Democratic Republic of Congo (DR Congo)	Declared state of emergency (March 24), closure of all borders, ban air travel and non-essential transportation between Kinshasa and 25 provinces (March 26), curfew and Kinshasa lockdown (March 26), and ban on intercity travels and later other towns (April 22, May 20). Global Monitoring (2020) and Tasamba (2020).
Burundi	Flights suspension (20 March), blockage of cargo transportation from East African Community (EAC) member countries (20 March), closure of borders with Rwanda and DR Congo, reopening border to cargo transportation (April 13). UN Migration Agency (2020)
Zambia	Shutdown of educational institutions and foreign travel restrictions (March 17), border closure (May 10), partial lockdown, partial closure of non-essential businesses, social gatherings ban, and suspension of cross border passenger and cargo transportation services. Nkomesha (2020) and World Aware (2020)
Zimbabwe	Declaration of state of disaster (March 20), prohibition of gatherings and national lockdown and curfews (March 30), and closure of non-essential services and business operations. KPMG (2020)
Mozambique	Cancellation of public events (March 19), international travel controls (March 20, April 1), public information campaigns (March 22), closure of schools (March 23), public gathering restrictions, workplace closure (March 30), closure of public transport, stay at home order, and restrictions on internal movement (April 1). Ask About (2020)
Cameroon	International travel control (March 13), closure of schools and public transport, and restriction on gatherings and internal movements (March 18), and workplace closure (May 1). Ask About (2020)

Low investment in agricultural mechanization exposed the bean value chain to the immediate effects of the pandemic. Bean production is SSA is labour-intensive and, therefore, the spread coronavirus and enforcement of social distancing, working from home, and restricted transportation are expected to affect labour-dependent operations such as planting, plant management, harvesting, threshing, and storage (Baudron et al., 2019; Nassary et al., 2020a, Nassary et al., 2020b). This poses a significant challenge on the transformation of smallholder agriculture from subsistence to market-oriented agriculture. Furthermore, cessation of movement was expected to cause significant disruption in food production and supply chains in major urban areas. As overall logistics slowed down, food safety and quality was negatively affected. Furthermore, the ban on public gathering and restricted open-air market operations compromised access to food in urban areas as a whole. In rural areas, COVID-19 containment measures impacted the supply of farm inputs such as bean seed and fertilizer, as well as access to extension services. These disruptions adversely affected bean production among rural poor and marginalized groups, cutting them off from output markets (Siche, 2020). Thus, the cumulative impact of COVID-19 may cause a severe setback to the achievement of Sustainable Development Goals (SDGs) that aim to eradicate hunger and poverty by 2030.

Consumption hubs in urban and peri-urban areas experienced food shortages because supplies from rural areas dropped due to mobility and transport restrictions. Additionally, the fear of being exposed to the virus and lockdowns constrained the supply of food to consumption hubs (Ezeh et al., 2020). The projected effect on food safety and consumption threatens to undo progress towards healthier and nutritious diets. Additionally, millions of people lost jobs, while businesses incurred losses, pushing millions of households into the risk of becoming poor (CGAP, 2020).

To mitigate the effects of COVID-19 on the agricultural and food systems, public and private sectors have come up with measures to ensure that agricultural value chain is not adversely affected (World Bank, 2020). For instance, at a time when some local markets were closing down due to travel restrictions, the **International Fund for Agricultural Development** (**IFAD**) helped connect farmers to buyers and also provided seeds and fertilizer to farmers in several countries in SSA (Rural21, 2020). In addition, the Pan Africa Bean Research Alliance - PABRA under the Alliance of Bioversity International and the International Center for Tropical Agriculture is working with their partners across different PABRA targeted countries to provided high iron and zinc certified bean seeds to farmers and extension services like the case in Nakuru, Kenya (Nchanji, 2020). The support is in recognition that over 70% of food consumed in the world is produced by smallholder farmers in rural areas and sold in urban areas (Ricciardi et al., 2018). While a continued assessment of the impact of COVID-19 on Africa's agriculture and food systems is ongoing, deployment of short-term solutions to production, distribution, and consumption hubs are underway to cushion smallholder farmers from income losses and urban population from risk of hunger.

This paper contributes to the already emerging literature on the impact of the COVID-19 pandemic on agriculture and food systems. Our focus is on the impact of COVID-19 on bean value chain in nine (9) countries in SSA: Kenya, Uganda, Tanzania, Burundi, DR Congo, Cameroon, Mozambique, Zambia, and Zimbabwe. We recognize that the published literature on COVID-19 effects on agriculture are reviews of literature, personal communications, news and blog content, and industry surveys and commentaries. Therefore, we provide a discussion of the immediate impact of COVID-19 on agriculture and food systems based on survey data collected in SSA.

### 1.1 Theoretical framework

**Fig. 1:**
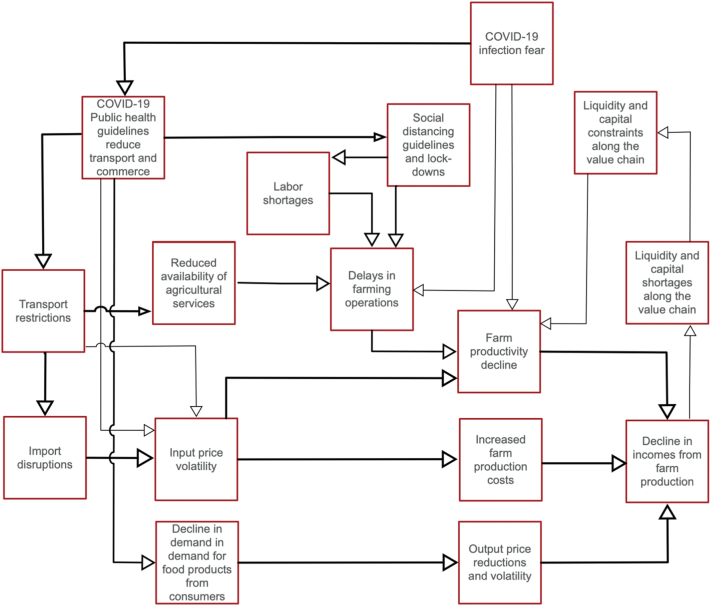
Indicators framework of the pathways of COVID-19 impacts on bean value chain in Sub-Saharan Africa. Adapted from Amjath-Babu et al. (2020).

The world has witnessed a couple of health-related crises in the last century. However, few pandemics have had significant range of effects like the outbreak and global spread of COVID-19 (CCAFS, 2020). The outbreak of influenza pandemics and severe acute respiratory syndrome (SARS) led to the formulation of several theoretical approaches explaining disease impacts on national, regional, and global economies. For instance, Brahmbhatt and Dutta (2008) used the economic epidemiology approach to highlight SARS dynamics, behavioural responses, and economic implications of behavioural responses, illness, and death. Another applicable framework is the cost of illness as used by Barratt et al. (2019) to account for the indirect cost implications of outbreak and spread of animal diseases. Nonetheless, besides health effects of coronavirus, containment measures have disrupted all sectors. The complexities of inter and intra-sectoral transmission of the COVID-19 related shocks left global agricultural stakeholders concerned about the impact of pandemic in developing countries, especially SSA countries. The pathways for the transmission are well-discussed by the indicator framework proposed by Amjath-Babu et al. (2020). In this study, we used the indicators framework to explain the pathways of the impact of coronavirus on bean value chain activities in SSA as shown in Fig. 1.

The indicators framework envisages direct and indirect pathways for the impacts of COVID-19 on agricultural value chains. COVID-19 outbreak and eventual spread across countries caused fear of infections, prompting governments to issue public health guidelines and containment measures, including restricted transport and commercial services. The pathways for transmission of COVID-19 impacts along the bean values chain due to transport restrictions are threefold. First, the restrictions reduce availability of agricultural services, causing delays in farming operations (Amjath-Babu et al., 2020). Second, transport restrictions disrupt importation of farm inputs such as seed, agro-chemicals, machinery, and fertilizer (Akiwumi, 2020; Willy et al., 2020). Third, COVID-19 induces transport restrictions cause logistical challenges, including partial closure of many agri-businesses, internal transport disruptions, and scaling down of international shipments (Amjath-Babu et al., 2020). Direct and indirect impact on transport restrictions cause input price volatility thereby increasing cost of bean production.

Furthermore, public health guidelines such as social distancing, stay-at-home orders, as well as lockdowns have two important effects on bean production. Social distancing limits hiring of labour and utilization of available household labour (Amjath-Babu et al., 2020) causing labour shortages. In addition, restricted movements of persons and transportation impacted on labour availability. The potential implications of these dynamics is the creation of labour shortages (Amjath-Babu et al., 2020), causing delays in farm operation.

High transportation cost, market restriction and public health concerns resulting in increased prices in urban areas amidst low purchasing power of the people. The delays in farming operations and the direct effect of fear for COVID-19 negatively impact farm productivity, leading to decline in farm incomes due to low sales revenues (Pan et al., 2020). Besides, input price volatility and decline in consumer demand result in an increase in bean production costs and output price reductions and volatility. The cumulative impact of these COVID-19 related effects is decline in liquidity and capital along the bean value chain because of the transmission of economic impacts to other agents. For instance, low grain volumes reduce business income for bean aggregators, network coordinators, informal and formal vendors, constraining them from maintaining critical stocks of grain and inputs (Amjath-Babu et al., 2020). The interactive negatively impact improvements in bean consumption and food and nutritional security in both rural and urban areas.

## 2 Methodology

### 2.1 Study area

**Fig. 2:**
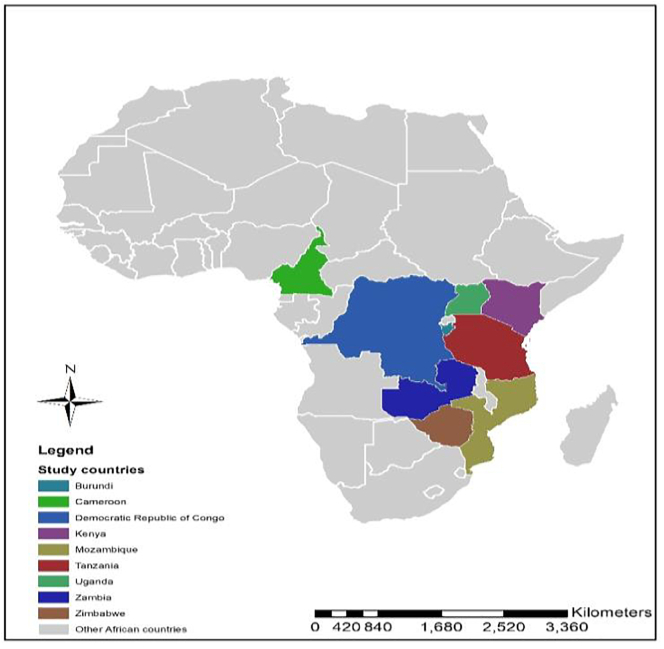
Map of study areas.

The study was conducted in 9 SSA countries between July and August 2020. Fig. 2 provide exact locations of the countries in the region.

### 2.2 Data collection

The data were collected using a mixed method from bean farmers, coordinators of bean programs, urban and peri-urban consumers, aggregators, processors, and mechanization service providers. The data were collected through web-based surveys, by sending links on WhatsApp and other mobile phone-based applications, and interviews. Face-to-face interviews were conducted in strict adherence to the COVID-19 health and safety measures and guidelines, including maintaining social distance, mask-wearing, and hand washing.

The data were collected through Survey Gizmo in English speaking countries. In French-speaking countries, the data were collected in French, then translated into English, and filled in the survey tool. The data were collected by National Agricultural Research Systems (NARS) partners. A total of 404 bean farmers, 5 coordinators, 4 female bean processors, 2 male agricultural equipment/machine dealers, and 431 urban and peri-urban consumers were surveyed. Furthermore, a web-based survey was administered to 10 bean grain aggregators in Eastern and Southern Africa. Table 2 provides the numbers and types of participants by country.

**Table 2:** Numbers and types of participants by country.

	Farmers	Aggregators	Coordinators	Processors	Machine dealers	Consumers
Kenya	41	1				35
Uganda	24	2		1		43
Tanzania	110	1	2	1	2	112
Burundi	41	1	1	1		37
DR Congo	42		1			45
Mozambique	36					36
Zambia	40	1				48
Zimbabwe	36	4	1	1		40
Cameroon	34					35
**Total**	**404**	**10**	**5**	**4**	**2**	**431**

### 2.3 Data analysis

The data analysis methods were overly descriptive. Exploratory data analysis of dataset variables was conducted using Excel and STATA. Statistics generated from raw data such as proportions and means were presented using data visualization methods, including multivariate non-graphical (cross-tabulations) and multivariate graphical methods.

## 3 Results and discussion

### 3.1 Effect of COVID-19 on the bean seed system

Ten Aggregators were asked about the business model they employed. All aggregators across countries (Kenya, Uganda, Tanzania, Burundi, Zambia and Zimbabwe) entered into contractual grain production agreements with farmer groups. In these agreements, eight out of 10 aggregators advanced seed credit to farmers. This is an indication of the crucial role played by aggregators in satisfying farmers' demand for bean seed. The result is an indication that aggregators need reliable and sustainable supply of grain of acceptable quality to sustain their business models.

The role of aggregators is critical in bean value chain in SSA. For instance, their innovative business models assist in overcoming seed delivery bottlenecks (Staatz et al., 2020). Nonetheless, COVID-19 is complicating their efforts in bridging seed supply gaps. For instance, seven aggregators in across eastern and southern Africa reported that COVID-19 had affected their distribution of seed to contracted farmers. The effect is much dire in Southern Africa countries than in Eastern Africa. Thus, the COVID-19 instigated disruptions of the bean seed system are likely to impact on seed multiplication, grain production, and access to quality seed (OECD, 2020a); crucial in the resilience of agricultural sectors and food systems.

Common bean is among the g crucial crop grown to improve incomes, food and nutrition security, and health outcomes of resource-poor smallholder households across SSA (Nassary et al., 2020a, Nassary et al., 2020b). Safeguarding Africa's food system requires the understanding of the impact of COVID-19 on production and multiplication of bean seed. Nevertheless, low farmer access to certified and quality seed is a major bottleneck to the potential contribution of common bean to the alleviation of poverty and food insecurity (Access to Seed Index, 2020; IFAD, 2018). There are indications that the COVID-19 pandemic may exacerbate the production and distribution of quality bean grain in SSA (Rubyogo et al., 2020). This finding underlines explanations provided by International Seed Federation (2020) and OECD (2020b), and Trade for International Development, 2020 about impact of transport restrictions due to the pandemic on seed sector.

This observation is underscored by coordinators of the bean research team in the different countries we targeted for this study. Analysis of data collected from coordinators shows that they projected the pandemic would reduce quantities of certified seed in 2020. For instance, the projected regional average quantity of certified seed is 881 metric tonnes against 901 metric tons pre-COVID-19. Although the change is minimal, it may exacerbate already existing constraints such as seed unavailability, affordability, and quality (Katungi et al., 2010; Munyaka et al., 2015). It may also thwart efforts that are already underway in improving seed production and distribution (Varshney et al., 2019).

Table 3 presents quantities of seed procured and supplied by bean coordinators within their networks pre-COVID-19 and during pandemic projections. The results show that COVID-19 will have an adverse effect on the production of certified seed in Burundi and Tanzania, with a projected level of seed production being below quantities produced in 2019. Furthermore, quantities of quality declared seed (QSD) are also expected to decrease in 2020 in Tanzania compared to other countries. The negative impact of COVID-19 on production Tanzania may be attributed to strict enforcement of COVID-19 restrictions of neighbouring countries such as Kenya, which host multiple seed companies that supply breeder and certified seed across the Eastern Africa countries (Access to Seeds Index, 2020). Movement of people critical to production, trade and sales may complicate access to seed. Border closures and delays in inspection and limited staff numbers means more time for clearance, slowing down overall supply chain functions. In contrast, seed production coordinators in Zimbabwe and the Democratic Republic of Congo projected that the quantities of certified seed in 2020 would be higher than those in 2019. This could be attributed to widespread interventions that will be in place after COVID-19 or lifting of restriction.

**Table 3:** Coordinators of bean research program estimates of the effect of COVID-19 on seed quantities in metric tons by country.

	2019				2020 projected			
	Certified	QDS	Breeder	Saved seed	Certified	QDS	Breeder	Saved seed
Tanzania	560	309	57		450	300	110	
Burundi	988	8	7	6528	850	35	5	5868
Zimbabwe	1840	1010	22	2000	1900	1100	21.00	3000
DR Congo	711		76	111	756		65.00	20

Note: Quality declared (QSD) seed in SSA is produced by farmers mostly under projects. They have to meet seed production and quality standards. Saved seed is recycled bean seed that is retained from grain harvested in previous seasons (s).

**Fig. 3:**
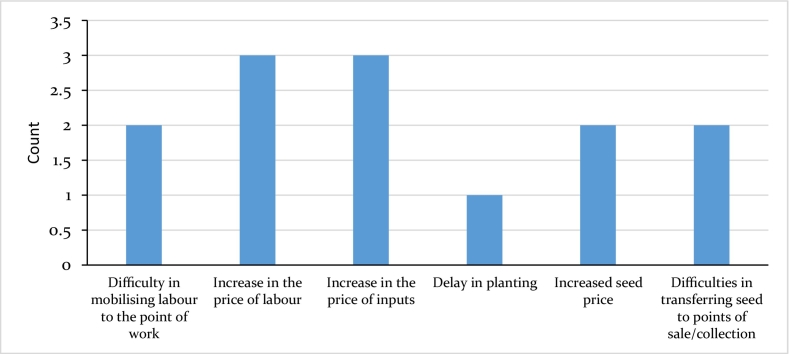
Count of bean coordinators responses to the effect of COVID-19 on seed production in 2020.

The bean network coordinators' projections of seed production is based on their opinion of the potential impact of the pandemic and climatic shocks on seed production in their respective countries, as well as production levels from previous season. Three out of the five coordinators (Democratic Republic of Congo, Zimbabwe, and Tanzania) reported that the pandemic had impacted seed production in 2020 due to increased cost of hired labour and inputs as shown in Fig. 3. They also identified the increase in seed prices and difficulties in mobilising labour and field-based inspections, as well as challenges involved in transferring seed to points of sale and collection of produced seed as challenges facing seed production enterprises in Eastern and Southern Africa. With the expected sharp rise in the demand for seed post-COVID-19, short-term to mid-term effects of the pandemic on seed production is likely to jeopardize seed assistance programs if nothing is done.

According to the CGIAR, it takes at least a season to produce and supply quality seed. As a result, as African farmers prepare to go back to their farms in the next cropping seasons, they are likely to struggle more in accessing and obtaining quality seed than they did before the pandemic (Ojiewo and Pillandi, 2020). Additionally, the bleak forecast in production of certified seed is expected to see farmers resort to increased use of ordinary grain as seed, thwarting progress made by on-going programs like Improving Bean Production and Marketing in Africa (IBPMA) the Cultivate Africa's Future Fund (CultiAF), and Tropical Legumes (TL) programs (Varshney et al., 2019) in promoting adoption of certified seed. The impact of the pandemic may go beyond affecting seed production by delaying certification of improved bean seed varieties.

#### 3.1.1 Effect of COVID-19 on access to agricultural services

The COVID-19 pandemic has been labelled as the biggest threat to the already weak agricultural sector in SSA (Ehui, 2020; Pais et al., 2020). The expected impact cuts across each node of the bean values chain. Focus on the possible impact of the pandemic has been on how grain production will respond to coronavirus containment measures implemented by various governments. The levels of restrictions differ from each country and region, and the impact of the containment measures are also expected to vary by country and region. To profile the short-term bean production challenges related to COVID-19, we asked farmers to state how they have been affected by the pandemic but being cognisant of the fact that they also face other production challenges.

Sampled farmers were asked to state whether bean production was affected by the pandemic. Analysis of their responses show that the pandemic has not affected bean production in Burundi and Cameroon. In contrast, every farmer (100%) interviewed farmer in Zambia, and 95% of their counterparts in DR Congo indicated COVID-19 impacted their bean production. Furthermore, half of the surveyed farmers in Uganda and 10%, 6%, and 3% of farmers in Kenya, Tanzania, and Mozambique reported that bean production was impacted by COVID-19, respectively. Nearly 31% of the farmers across the nine countries reported that COVID-19 had impacted bean production. More farmers in the Southern African region (55%) than in the Eastern Africa region (24%) reported that the pandemic impacted bean production.

It is noteworthy that the Southern and Eastern Africa farmers had higher chances of reporting the effects of the pandemic on production; because the first reported cases of COVID-19 and government restrictions on movement and lockdowns happened at the beginning of the February-April 2020 cropping seasons in eastern Africa and October to February cropping seasons (e.g. in Mozambique, DR Congo, Uganda and Kenya). These results reveal that the possible impacts of the pandemic on the production of beans and other crops are also dependent on cropping seasons rather than just the restrictions imposed by governments.

Comparison of impact by country shows that bean production in Tanzania is impacted by the effect of the pandemic on the cost of labour (Table 4). In contrast, almost 67% of the surveyed farmers in Kenya reported that high cost of hired labour and farm inputs, and low demand of bean grain in the market were significantly affected. Farmers in Mozambique, DR Congo, and Zimbabwe reported seed unavailability as COVID-19 a related impact on bean production. Almost half (49%) of sampled Zambian farmers reported that COVID-19 had caused grain transport challenges from farms to points of sale (Table 3). These results indicate the diverse impacts of the pandemic on bean production, which are possibly sporadic depending on restrictions and seasonality issues. For instance, agriculture in SSA is labour intensive (both farmer's own and hired labour force). Thus, social distancing restrictions disrupted labour availability and cost (Schmidhuber et al., 2020). On the other hand, countries with imposed partial or full lockdown could have disrupted distribution of seed and other farm inputs, impacting on bean production.

**Table 4:** Proportions (%) of farmers' responses on the effects of COVID-19 pandemic on bean production by country.

COVID-19 effects	Kenya	Mozambique	DRC	Tanzania	Uganda	Zambia	Zimbabwe
High prices for hired labour	22.22		1.22	63.64		1.28	
Higher prices for inputs	22.22		19.51			3.85	31.03
Low demand in the market	22.22			9.09	21.43	2.56	12.07
Fertilizer unavailability	11.11		1.22		14.29	1.28	
Low price in the market	11.11		6.1	9.09	28.57		5.17
Difficulties in transporting the harvest to the point of	11.11			9.09	14.29	48.72	18.97
Seed unavailability		100.00	47.56			1.28	32.76
Delay in planting			15.85			2.56	
Delayed harvest			8.54		7.14	1.28	
Difficulty in accessing agronomic information				9.09	14.29	37.18	

**Fig. 4:**
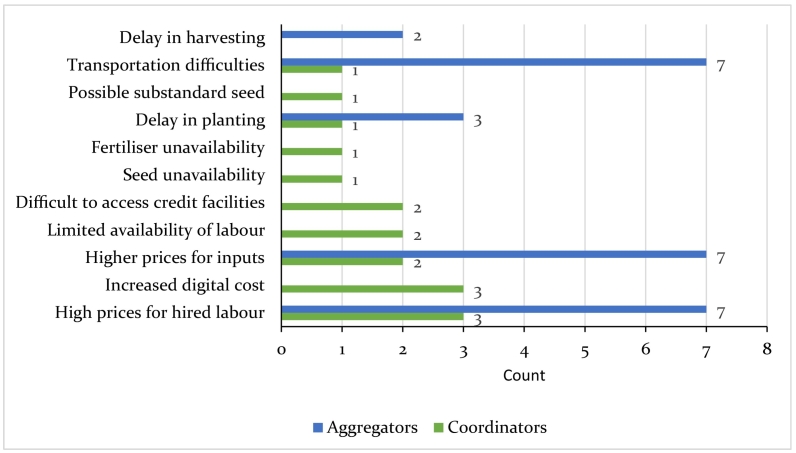
Count of coordinators and aggregators responses to the effect of COVID-19 on bean production.

The bean program coordinators and aggregators were also interviewed on the effects or potential threats of the pandemic on bean production in 2020. Their responses corroborate the effects reported by farmers. They observed that the COVID-19 led to high cost of hired labour and farm inputs (Fig. 4). Additionally, aggregators and coordinators identified constraints in transporting bean harvest from the farm gate to the points of sale. In the short-term, bean network coordinators and aggregators also reported that COVID-19 is impacting bean production by causing delays at the start of planting and harvesting, seed availability and difficulties in accessing credit. Responses from bean network coordinators in Tanzania, Burundi, and DR Congo indicated that increase in frequencies of climate-related occurrences such as rains, floods, drought, and high temperatures affected or were anticipated to decrease bean production in the two 2020 cropping seasons. Furthermore, the bean network coordinators identified the increase in pests and diseases and post-harvest losses as some of the major threats to an increase in bean production in 2020.

The immediate impact of COVID-19, coupled with the adverse effects of climate change as reported by aggregators and coordinators, threaten to undermine the improvement in bean production in SSA. Furthermore, seed shortages as a result of the pandemic are expected to have ripple effects along the value chain. The first effect is on bean grain production. Based on their previous season's production, network coordinators were asked to project volumes of bean grain that would be produced in 2020. Table 5 compares the estimates of bean grain produced in 2019 to the projected quantities in 2020. The combined result shows that the average quantities of bean grain produced across the countries will be 435,000 metric tons in 2020 compared to 488,000 metric tons produced in 2019. According to bean network coordinators projections presented in Table 4, grain production in Tanzania, Burundi, and DR Congo are expected to decline by 10.9% in 2020 compared to the 2019 production levels. However, grain production in Zimbabwe and Mozambique in 2020 are expected to be relatively above the 2019 production levels.

**Table 5:** The average quantities of bean grain produced in 2019 and projected grain quantities in 2020 as estimate by coordinators.

Country	2019	2020 projected
Tanzania	1,200,000	1,050,000
Burundi	434,000	410,000
Mozambique	75,000	80,000
Zimbabwe	12,650	13,000
DR Congo	6050	6000
Pooled	487,950.00	434,833

#### 3.1.2 Effect of COVID-19 on bean grain business, distribution, and trade

The impact of COVID-19 in SSA goes beyond seed and grain production. The downstream distribution and demand for bean grain are also expected to be affected by the transmission of COVID-19 from seed and grain production to aggregation and trade. Presently, the concern is the breakdown in the supply chain of bean grain to aggregators, small and medium bean enterprises, and processors. The small and medium enterprises (SMEs) are expected to be deeply affected by social distancing and trade restriction because of the informal nature of their trade with low resilience to economic and non-economic crises. We interviewed aggregators and trader of bean products about the effects of the pandemic on their bean businesses.

Aggregators in Zimbabwe, Uganda, Kenya, Zambia, and Tanzania noted that they face insurmountable challenges since the first cases of COVID-19 were reported in their respective countries. Aggregators reported that the pandemic has reduced sales volumes, due to the closure of grain markets. Most aggregators sell grain to informal vendors who have also been affected by almost complete closure of business such as hotels and schools where most beans were absorbed. In addition, aggregators are facing a challenge of low grain prices due to decreased demand for bean grain in the market as most traders have scaled down business operations. Third, some aggregators reported slow movement of grain due to border closure and travel restrictions as a result of partial lockdowns in some of the countries in Southern and Eastern Africa.

Countries in Eastern and Southern Africa responded to the unprecedented outbreak on the continent by imposing varying degrees of restrictions that affected cross-border trade. A majority of the aggregators' highlighted transportation costs and an increase in logistical costs, as crucial impact of the pandemic on beans cross-border trade. Additionally, COVID-19 has increased costs related to storage of beans. The cross-border bean trade is expected to decline in Eastern and Southern Africa with COVID-pandemic as reported by bean network coordinators in DR Congo and Burundi. This contradicts projections provided by aggregators who were optimistic that the volume of grain traded this year would increase. Notwithstanding projection, contradictions by aggregators and coordinators, observers note that the COVID-19 pandemic is already causing direct and indirect reduction in volumes of trade in most agricultural commodities in the regions.COVID-19 will contribute to a decline on dry bean trade quantities that have been increasing since the last quarter of 2019 (East Africa Cross Border Trade Bulletin, 2020).

**Fig. 5:**
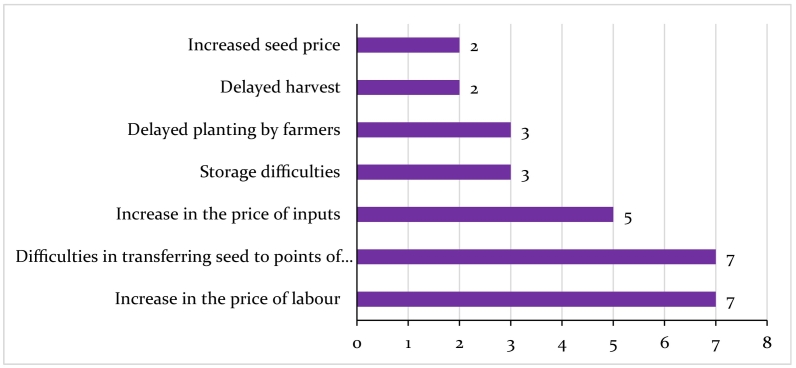
Proportions of aggregators' responses to the adverse effects of COVID-19 pandemic and government measures on bean trade.

The pandemic and government restrictions also resulted in increased cost of inputs, delayed planting and limited storage spaces (Fig. 5). Moreover, some aggregators reported that cross-border trade is impacted because of lack of bean grain resulting from high seed prices and delays in grain harvesting. Seed system activities have been hampered, causing delays in bean planting. The domino effect is the automatic shift in harvesting dates, which negatively impacts cross-border bean trade.

Four processors of bean products interviewed from Zimbabwe, Uganda, Burundi, and Tanzania indicated that their main processed bean product was flour. Analysis of their responses to the survey questions indicate that except for the Zimbabwean processors, the others were optimistic that they would be an increase in the volumes of bean grain to be processed this year compared to last year. For processors in Burundi and Tanzania, their positive outlook is attributed to relatively low impact of COVID-19 on bean production as reported by farmers. The Ugandan processors' positive projection of processed bean quantities is unexpected due to coordinators and farmers, indicating that the pandemic has relatively less negatively impacted grain production. While the processor in Zimbabwe reported that the sale of processed products was poor pre-COVID-19 and would remain unchanged in 2020, Ugandan and Tanzanian processors indicated the sale of processed flour was average before the first cases of COVID were reported. The processors in Burundi and Tanzania were optimistic that sale of processed bean product would remain good and average respectively predicting from the good performance in 2019, the Ugandan processor was less optimistic by projecting sale of the processed product will be poor in 2020.

All processors confirmed that government measures during COVID-19 is affecting their businesses. Sales have decreased due to low consumption of bean products. Consequently, their businesses are operating below capacity. For instance, the processor in Zimbabwe reported that the business is only operating at 25% of its usual capacity, while processors in Uganda and Tanzania indicated that they are operating at half of their capacity. The processor in Burundi reported that the business is operating at 75% of its capacity. These results are further underscored by aggregators, who reported that their businesses slowed down during the pandemic. While three out of four bean aggregation businesses in Zimbabwe, one in Uganda, and those in Tanzania, and Zambia operated at 75% of the pre-COVID-19 levels, those in Kenya, Burundi, and one in Uganda operated at 25% of their capacities. Another aggregator in Zimbabwe reported that the business operated at half its capacity. .

#### 3.1.3 Effect of COVID-19 on bean consumption and food security

Prior to the outbreak of the COVID-19 pandemic, the African agricultural and food system could not sustainably meet the food demands by the population. The continent relied on agricultural imports to bridge the food supply and demand gap. As of 2019, the African countries imported between US$45–50 billion worth of food commodities (Pais et al., 2020). These statistics provide a clear indication of the role of trade in provision of food in Africa (Rubyogo et al., 2020). The openness of agricultural and food markets means shocks in the international markets may have a bearing on food supply in the region. With this in mind, governments, development partners, and agricultural value chain actors speculate that the pandemic, accompanied by restrictive measures, would disrupt agricultural trade, leading to dire implications on the food system. Information from farmers and consumers about their patterns of food consumption before and during COVID-19 confirm this.

**Fig. 6:**
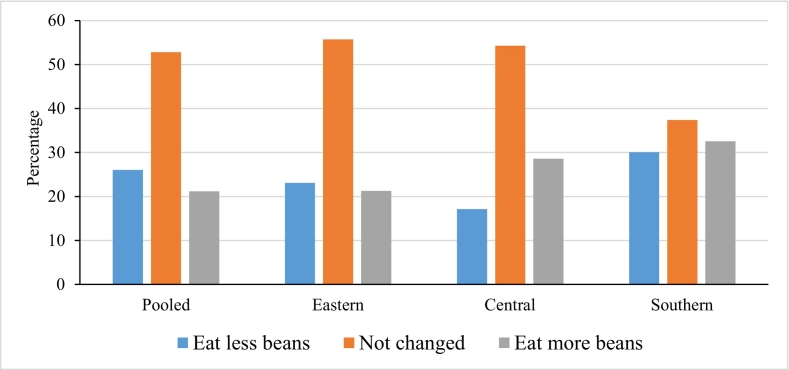
Changes in bean consumption patterns in urban and peri-urban areas since COVID-19 by region.

Fig. 6, above, summarizes urban and peri-urban patterns of bean consumption in three regions of Central, Eastern and Southern Africa. Aggregated results of all surveyed consumers show that more than half (53%) of them have not changed bean consumption patterns during the pandemic. More consumers in Eastern Africa have not changed their bean consumption patterns during the pandemic compared to those in Central (54%) and in Southern (37%) Africa regions. The results displayed in Fig. 6 suggests that weakly bean consumption during the pandemic has relatively been affected in Southern Africa compared to the other two regions with the exception of Zambia that had good harvest pre-pandemic (Ehui, 2020), food production in Zimbabwe and Mozambique were adversely affected by climate-induced shocks, putting pressure on food systems (Akiwumi, 2020; Ayanlade and Radeny, 2020; Paul, 2020). Thus, COVID-19 is exacerbating the already dire food insecurity situations in the sub-region.

**Fig. 7:**
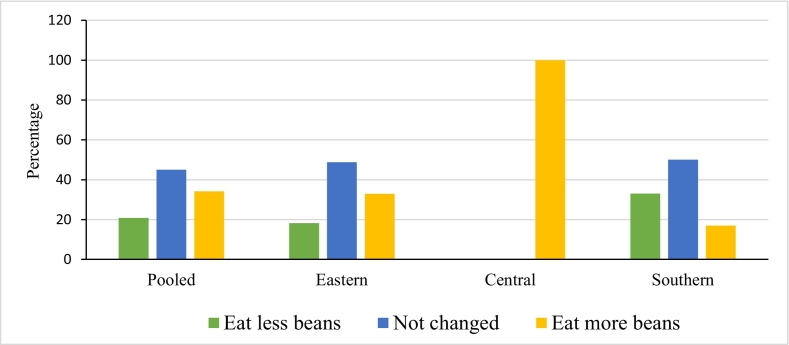
Changes in bean consumption patterns in rural areas since COVID-19 by region.

In contrast to bean consumption pattern by urban and peri-urban consumers, farm level patterns of bean consumption were affected by the pandemic as indicated in Fig. 7. While nearly 45% of farmers surveyed across the region reported household bean consumption patterns have not changed during the pandemic, 55% observed that consumption patterns have changed. For instance, 21% of the surveyed rural households ate less beans compared to 34% of who ate more beans per week during the pandemic. By region comparison of the results indicates that all surveyed farmers in central Africa are eating more beans during COVID-19 compared to their counterparts in Eastern Africa (33%) and Southern Africa (17%). The proportions of farmers that eat less beans per week during COVID-19 is more in Southern Africa countries than in Eastern Africa. This could be attributed to the fact that most farmers in Zambia and Zimbabwe reported that the pandemic had adverse effects on their bean production.

The changes in consumption are expected to cause changes in the frequency of bean consumption at the household level. Table 6, below, shows the frequencies of bean consumption by the surveyed bean consumers in urban and peri-urban areas have been adversely affected in Southern Africa countries than in other regions. For instance, the proportions of consumers that ate beans once per week in Zimbabwe increased from 44% pre-COVID-19 to 62% during COVID-19. A similar trend is shown in Zambia. Bean consumption by urban and peri-urban consumers almost remained unchanged in Cameroon, possibly because of the almost little impact of the pandemic on bean production as earlier reported. In Eastern Africa, the pandemic appears to have increased frequency of consumption in urban and peri-urban areas from twice or once per week before the pandemic to almost daily and more than three times. In Kenya, Tanzania, and Burundi, urban and rural bean consumption frequency appear to have slightly reduced during the pandemic. In contrast, DR Congo appears to be the most affected country in Eastern Africa, with about 20% of the households consuming beans every day during the pandemic compared to 31% that consumed beans before the pandemic COVID-19 (Table 5). The frequency of bean consumption in Uganda has increased, with the proportions of everyday consumption increasing from 2% pre-COVID-19 to 27% during the pandemic. On the other proportions of households that consumed bean once per week reduced from 59% before the pandemic to 18% during the pandemic.

**Table 6:** Proportions of consumers' frequency of bean consumption a week before COVID-19 and During COVID-19 by country.

	Before COVID-19					During COVID-19				
	Everyday	> thrice	Thrice	Twice	Once	Everyday	> thrice	Thrice	Twice	Once
Burundi	67.57	8.11	10.81	13.51		40.54	10.81	24.32	24.32	
Cameroon	2.86	8.57	5.71	22.86	60.00	2.86	11.43		25.71	60.00
Kenya	2.86	14.29	25.71	34.29	22.86	2.86	5.71	20.00	40.00	31.43
Mozambique		2.78	11.11	27.78	58.33		2.78	16.67	36.11	44.44
DRC	31.11	17.78	31.11	15.56	4.44	20	15.56	20	24.44	20.00
Tanzania	5.36	46.43	16.07	25.00	7.14	6.25	53.57	13.39	19.64	7.14
Uganda	2.27	6.82	11.36	20.45	59.09	27.27	15.91	15.91	22.73	18.18
Zambia		4.17	10.42	22.92	62.5		4.17	16.67	31.25	47.92
Zimbabwe		12.82	10.26	33.33	43.59		10.26	2.56	25.64	61.54

Furthermore, the anticipated immediate impact of the pandemic on food markets in urban and peri-urban areas cannot be overstated because of challenges in transportation of food from production hubs (International Trade Centre, 2020). Results in Table 6 indicate that the consumption of all food products remained unchanged during the pandemic compared to their consumption during the pandemic, except fish and meat consumption in Central Africa. The salient finding from the result presented in Table 6 indicate that the pandemic could be cause logistical bottlenecks that will affect in country availability and cross-border trade for those countries that heavily rely on intra-regional and inter-regional trade for food supplies. Another salient finding indicates that consumers in Central Africa (Cameroon) are consuming more of other legumes and vegetables now than before the pandemic, but none of them is consuming meat or fish. In addition, meat and fish consumption in Southern and Eastern Africa countries declined during the pandemic. Increased vegetable consumption in Central, for example, could be attributed to the promotion of consumption of vitamin-rich foods as a strategy of boosting immunity against coronavirus infection. Similar results are reported by farming households that were surveyed in the nine countries (see Tables 7).

**Table 7:** Proportions (%) of consumers that ate different types of food before and during the pandemic by region.

	Before COVID-19				During COVID-19			
	Pooled	Eastern	Central	Southern	Pooled	Eastern	Central	Southern
Bean	89.54	88.89	74.29	95.12	80.1	89.05	68.57	61.79
Cereals	86.99	83.57	62.86	97.56	75.77	82.12	60	66.67
Roots and tubers	76.53	72.63	71.43	81.3	66.33	71.17	48.57	55.28
Other legumes	56.63	53.65	25.71	72.36	38.52	45.26	34.29	26.83
Vegetable	87.24	89.78	2.86	100	85.71	85.04	11.43	95.12
Fruits	63.27	60.22	5.71	76.42	47.7	57.66	2.86	28.46
Meat	68.11	66.42	100	86.99	43.88	49.27	0	38.21
Fish	64.03	60.22	100	87.8	45.66	44.89	0	52.85

The results in Table 7, Table 8 indicate the immediate impact of COVID-19 on food security. For urban and peri-urban consumers, The differences in proportions of food types consumed pre-COVID-19 and during the pandemic were significantly different except for meat and fruit consumption in Southern and Central Africa sub-regions respectively. There were statistically significant reductions in consumption of diverse varieties of food by farmers in Eastern and Southern Africa, except for bean and other legumes, and meat in latter region. The decline in proportions of households consuming diverse food varieties threatens to exacerbate food insecurity problem in SSA. The reductions in food types consumed could be attributes to reduced farm incomes and millions of job losses resulting in low purchasing power and food demand (Amjath-Babu et al., 2020; Mbewa, 2020). These represent losses of income and livelihoods for those involved in the bean value chain. Additionally, the pandemic may have forced farmers to either consume seed as grain in order to cushion themselves against reduced access to food. These scenarios are a major bottleneck to the progress towards ending hunger in SSA.

**Table 8:** Foods consumption by farmers in before and during the pandemic by region.

	Before COVID-19				During COVID-19			
	Pooled	Eastern	Central	Southern	Pooled	Eastern	Central	Southern
Beans	97.77	97.29	97.06	99.11	89.85	98.06	97.06	68.75
Cereals	94.55	93.02	94.12	98.21	80.2	83.72	91.18	68.75
Roots and tubers	81.44	79.07	85.29	85.71	59.16	74.42	38.24	30.36
Other legumes	50	32.95	82.35	79.46	28.22	25.97	82.35	16.96
Vegetables	88.12	85.66	70.59	99.11	85.15	85.27	82.35	85.71
Fruits	59.9	61.63	5.88	72.32	46.29	60.08	38.24	16.96
Meat	62.87	56.59	2.94	95.54	46.29	39.15	2.94	30.36
Fish	58.17	54.65	0.00	83.93	36.14	44.19	0.00	28.57

#### 3.1.4 Government support and strategies employed by farmers, consumers, and aggregators in response to the pandemic

Like other economic and non-economic crises, individuals, households, businesses, and governments responded to the pandemic in various ways. The study was to establish how farmers, urban and peri-urban consumers, aggregators, and processors responded to the pandemic. Urban agriculture contributes to improved food and nutritional security in most cities across the world. Thus, for consumers owning home garden, we asked them to state whether they made any changes to home garden during the pandemic. The salient finding is that most urban consumers who increased the size home garden for with view of supplementing household food supplies during the were able increase bean consumption. Consumers who increased size of home garden as a pathway for reducing financial burden on food supplies were able to maintain/stabilize the level of bean consumption before the pandemic. On the other hand, those that increased home garden for fresh and healthy foods ate less bean in during the pandemic. The results imply that access to food during the pandemic mattered most in reducing food-consumption impacts of the pandemic than access to fresh and healthier food. In other words, these results are also plausible because kitchen gardens may have enabled urban and peri-urban consumers to overcome reduced availability challenges due to restricted transportation or high grain prices. Besides expansion of kitchen garden, a few urban and peri-urban households in Kenya, DR Congo, and Uganda received relief food from governments.

The onset of COVID-19 in SSA found many countries in the region already grappling with the challenge of boosting the agricultural sector from the adverse effects of climate change, pest and diseases, and civic insecurity issues. For instance, the Horn of Africa and part of Eastern Africa was experiencing locust invasion, prolonged droughts, and conflicts that were projected to constrict the expansion of agricultural production (Blanke, 2020). Agriculture in the region is dominated by smallholder farmers that are disproportionately affected by the adverse effects of climate change and internal conflicts. The pandemic represents a significant challenge to agriculture and food production in the region. Cognisant of the pandemic's effects on agriculture, governments across the region identified priority areas that commanded immediate address to improve the resilience of the sector to COVID-19 related effect. The farmers, processors, aggregators, and coordinators were aware of their governments' support and the benefits derived from such support.

Farmers in Cameroon, Mozambique, DR Congo, and Burundi were hardly aware of government support to the agricultural sector during the pandemic. Nearly one-quarter of the surveyed farmers in Kenya, 33% in Tanzania, 12% in Uganda, 58% in Zimbabwe, and all farmers in Zambia were aware of government support in the agricultural sector during the on-going pandemic. For instance, farmers in Kenya, Uganada, and Mozambique indicated that they benefited from favourable seed and fertilizer prices in government supported programs. Some of the government supported programs included e-voucher initiative in Kenya, special facilitation and support of transportation of planting material by Ugandan government, Farmer Input Support Programme (FISP) and the National Food and Nutrition Commission (NFNC) in Zambia. Nearly 39% of the farmers aware of government support were already beneficiaries of such programs. Approximately 9%, 10%, 67%, 88%, and 5% of the surveyed farmers in Kenya, Tanzania, Uganda, Zambia, and Zimbabwe respectively were already beneficiaries of such government programs. Majority of the beneficiaries mentioned that they already enjoyed government support for food crop production through access to free seed and subsidized fertilizer. This happened in countries where governments included beans as part of food relief packages. Other benefits mentioned by the farmers include seed price subsidies, increased access to labour possibly due to closure of schools and non-farm business, supply of irrigation water, and facilitation to access digital agronomic information about bean production.

Concerns about the impact of the pandemic on food distribution and consumption can be addressed by supporting downstream activities such as aggregation, trade, processing, and bean coordination programs. These stakeholders are not only important in facilitating the movement of food products along the value chain, but also key supporters in upstream activities such as grain and seed production, and provision of market information. Aggregators are critical in grain bulking thus, supporting them through public-private partnerships in the face of the pandemic is crucial in addressing the disruption in grain supply and trade. Nonetheless, the aggregators in the seven out of the nine countries mentioned that they had not received business contracts to provide grain for food emergency supply purposes. While aggregators who received government business contracts to provide relief food during the pandemic supplied less than 1000 metric tons of bean grain.

Aggregators in Zambia, Uganda, Kenya, and Burundi indicated that there were no government incentives supporting aggregators and traders, their counterparts in Tanzania acknowledged government support during the pandemic. Tanzanian aggregator reported that they benefited from the government's fuel price subsidy and favourable financing opportunities during the pandemic. The three aggregators in Zimbabwe mentioned that they benefited from new policies and incentives on certified seed production, favourable financing opportunities, and new market opportunities provided by the Zimbabwean government since the beginning of the pandemic.

Furthermore, bean research coordinators in Burundi, Democratic Republic of Congo, Tanzania, and Zimbabwe, reported that their governments had implemented various incentives to support bean production during the pandemic. For instance, according to the bean research coordinator in Burundi, the Burundian government provided favourable prices on fertilizer, introduced new production subsidy policies, and ensured reasonable price of bean grain in the market. In Tanzania, the government also ensured reasonable price of beans in the market, while the Zimbabwean government ensured availability of water for conservation agriculture through irrigation, provided new production subsidy framework for agricultural inputs, ensured that bean was reasonably priced in the market., and also increased demand for beans in the market. According to the surveyed bean research coordinators, these interventions cushioned farmers by lowering production costs and increased access to agronomic information.

None of the processors and mechanization providers in Tanzania nor processors of bean products were aware of any government programs to support their businesses during the pandemic. Additionally, processors in Tanzania, Burundi, Zimbabwe, and Uganda reported that the government did not purchase any processed product from them as relief food. However, at the business level, mechanization providers in Tanzania have created the video user manual that they share with farmers on social media whenever they buy threshers. In addition, one of the mechanization dealers in Tanzania also indicated that they are registering farmers for mechanization through mobile phone and payments are done through mobile money transfers. On the other hand, aggregators from Tanzania, Burundi, Kenya, Uganda, and Zimbabwe mentioned that they have developed innovative ways for doing business as a result of the pandemic. Some of the innovations adopted by aggregators to conduct business amidst the pandemic are using digital and technical innovations such as social media marketing to reach the buyers.

### 3.2 Discussion

The pandemic has not only caused an ongoing crisis in the health care sector but has also had immediate and widespread impact on Africa's agriculture and food systems (Ayanlade and Radeny, 2020). Although some countries in SSA have begun easing the restrictions put in place to curb the spread of the virus, the impact on bean production, distribution, and consumption remain in the short-term and long-term. This is because recovery from the immediate impact of the pandemic will be slow and uncertain. Direct and indirect effects across the bean value chain will still be experienced as the most African economies struggle to revert to their pre-COVID-19 growth level.

The results presented in this paper show that impact of the pandemic on bean production are inextricably linked to pre-existing bean value chain situations. In other words, the pandemic has exacerbated the challenges the agricultural sector faced before COVID-19. First, the immediate impact of the pandemic is the disruption of the supply of essential yield-increasing inputs such as fertilizer and certified seed (AU, 2020; OECD, 2020c). The consequence of these disruption on input supply is due to an increase in input prices. Furthermore, the results reveal challenges in logistics and transport resulting in a glut supply at bean production hubs, leading to low demand and low farmgate/rural prices across the regions (GAIN, 2020; International Trade Centre, 2020; OECD, 2020c). As reported by all bean value chain stakeholder in the nine countries, the pandemic and government movement bans and increase in public transportation costs have caused reduction in affordable and available labour and mobility (Stephens et al., 2020). These impacts affect family-based bean farming enterprises whose direct effect is a drop in household income, individual and household purchasing power, and limited access to traded food. The indirect effect of bean production disruptions is reduced food availability and steady supply.

The immediate impact of the strict public health policies such as quarantine after local, national, and regional travel has undoubtedly dropped the profitability and performance of Small Medium Enterprises (SMEs) such as grain distributors, aggregators, and transporters (Béné, 2020). Also, the risk of exposure to the disease has almost halted business operations for both formal and informal retailing SMEs, vendors, and processors. Similar results were reported by Mahajan and Tomar (2020) who explained that a drop in supply of fresh food following imposition of first lockdown in India. These shocks contribute to increased food prices and disrupted access to food outlets for consumers. This finding is in line with Narayanan and Saha (2020) and Varshney et al. (2020) who reported that lockdowns increased prices of pulses and wheat in India respectively. Furthermore, consumers' incomes are lost as a result of the farm closure and non-farm business. These imply that consumers, food and non-food actor, are impacted adversely. The consequences of the pandemic on production, distribution, and consumption of beans and other food items is a threat to the achievement of SDGs targeting eradication of hunger and poverty in SSA (FAO, 2020). Thus, the pandemic is expected to increase food and nutritional insecurity in the region in the short-term and long-term.

## 4 Conclusion and policy implications

The outbreak and subsequent spread of the pandemic has not only imposed a significant burden on the global health care system but has also posed a challenge in all segments of agriculture and food systems across the world. The immediate impact of the pandemic was initially projected to destabilize the agricultural sector in developing countries as a result of their low resilience to production shocks and a myriad of other bottlenecks that existed before COVID-19. The magnitude of the immediate impact of the pandemic varies depending on the public health policies from various governments in combating the spread of the virus. Social distancing, movement restrictions, border closure, quarantine, are some of the shocks to production, distribution, and consumption of agricultural produce. As a result, we focused on the immediate impacts created by the pandemic on food systems in SSA.

The results reveal that bean value chains in Eastern and Southern Africa face production bottlenecks linked to the disruption caused by the pandemic and government restrictions. Farmers, aggregators, and bean research coordinators confirmed that access to labour and input supplies were greatly disrupted, transportation of farm produce was impacted, price of agricultural inputs skyrocketed, and grain demand plummeted, resulting in projected production losses. Secondly, the shutdown of most economies in Eastern and Southern Africa disrupted the bean supply chain with aggregation, distribution, and processing of bean grain greatly affected. Aggregators are experiencing logistic challenges in accessing production hubs and transporting the produce to points of sale. SME processors are affected by labour shortages and high cost on available labour. Furthermore, bean consumption patterns have moderately changed, especially in urban and peri-urban areas due to market closure and restricted transportation, and high food prices.

However, the immediate impact of the pandemic and government restrictions to curb the spread of the virus are further magnifying the already existing agricultural production challenges in the region. Prior to the pandemic, the bean value chain in SSA was already faced with low utilization of yield-enhancing technology (improved seed and fertilizer). Additionally, the sector was grappling with locust invasion coupled with adverse effects of climate change and weather variability. Thus, the pandemic exacerbated the effects of the challenges that the agricultural sector in the region was exposed to.

Government policies for combating the impact of the pandemic on bean values chain are inextricably linked to the pre-COVID-19 challenges that existed in the bean production, distribution, and consumption. The disruptions caused by the pandemic are simultaneously affecting the bean supply chain. The implications for these findings are that the challenges are complex and pose an additional threat to the achievement of sustainable development goals of ending hunger and poverty in SSA. The significance of these descriptive findings is that in the short-term to medium term, disruption of the bean values chain will create a temporary surplus for bean producers and deny them the purchasing power and improvements in food security. In contrast, the pandemic will create food shortages for consumers because of limited access and availability of food. The pandemic also threatens to halt operations of business operators, especially SMEs in bean processing. For these reasons, these challenges need to be addressed.

Governments could play a significant role in supporting the needs of smallholder farmers, traders and other actors. First, existing input subsidy programs and relief packages need to be strengthened to ensure adequate supply of affordable inputs to most vulnerable bean farming households. This would promote and fast-track recovery of bean production. Second, food security for any country during a pandemic is of utmost importance, the governments should prioritise agriculture as an essential service during pandemics and declare farm and food workers as essential service providers. Additionally, public-private partnership should support farmers and producer organizations by identifying seed and grain collection and aggregation/storage centres and, where possible, offer warehouse receipts and vouchers to facilitate access to both input and output markets. Furthermore, insurance, financial, and tax incentives, as well as digital capacity building programs should be extended to cushion and enhance resilience of producers and distributors in the agricultural value chain. Governments should also consider a win-win situation for all bean value chain actors by encouraging and supporting bulk purchasing, onward distribution of grain, and controlled pricing. These would not only reduce the impact the pandemic production and bean business, but also improve food and nutritional security by keeping price increase at minimum.

Furthermore, governments and National Agricultural Research Systems(NARS) should support SMEs in developing innovative business models that enable them to cope with the effects of any ensuing pandemic. For instance, support should involve technical capacity building of SMEs in the use of digital businesses models. Lastly, vulnerable consumers and traders should be supported by keeping food markets open and safe during pandemics, supporting food transportation and distribution, and offering food price subsidies.

## Ethical consideration

The aim of the study was clearly explained to all respondents. Permission was sought verbally before data collection. Respondents were informed of their right to stop the interview or refuse to participate in the research. Few respondents opted out during this study.

## Declaration of Competing Interest

The authors declare that they have no known competing financial interests or personal relationships that could have appeared to influence the work reported in this paper.
